# Hypothetical errors and plateaus: A response to Newman

**DOI:** 10.1371/journal.pbio.3000076

**Published:** 2018-12-20

**Authors:** Kenneth W. Wachter

**Affiliations:** Department of Demography, University of California, Berkeley, California, United States of America

## Abstract

Newman questions recent claims about a plateau in mortality rates for Italians beyond age 105 on the basis of a hypothetical model. His model implies implausibly high error rates for extreme ages. For individuals over 110, for whom birth certificates have been collected, the form in which Italian births were registered precludes the kinds of clerical errors in year of birth that Newman assumes.

## Abstract 2

This Formal Comment responds to an article by Saul Newman, and maintains that data errors do not explain mortality plateaus.

This paper is a response to the comment by Saul Newman [1], which asserts that “the mortality plateau in Barbi and colleagues [2] can be generated by low-frequency randomly distributed age misreporting errors.”

Newman does not discuss the actual observations on which Barbi and colleagues rely, which pertain to 3,836 deaths over the the age of 105 in new specially collected Italian data from 15 cohorts. Instead, Newman offers a hypothetical scenario and shows that a certain stylized form of age misreporting can generate the appearance of a plateau from underlying log-linear hazards. This general fact has been known for nearly a century and is mentioned by Barbi and colleagues. It is only relevant to the question of plateaus beyond 105 if a specific sufficient form of age misreporting beyond 105 can be shown to be likely in the new data. Newman presents no specifics to support such a case.

It is easy to work out predictions from Newman’s hypothetical model for the proportions of true and erroneous ages at any reported extreme age *x*. In the model, a proportion *p*/4 of the population are 10 years younger than their reported ages and similar proportions for five years younger, five years older, and 10 years older, with *p* = 0.005 and *p* = 0.001 for Newman’s Figure 1. Let *A* be the number of survivors to true age *x* − 10. These contribute *A* × *p*/4 erroneous *x*-year-olds. (At extreme ages, other error contributions are too small to matter.) To predict true *x*-year-olds, multiply *A* by the log-linear model value for survivorship from age *x* − 10 to *x* and multiply by 1 − *p*. With the log-linear parameters that Newman is using, only 0.000064 of 100-year-olds survive to a true age of 110. Thus, under Newman’s assumptions, almost all the purported 110-year-olds would be predicted to be erroneously reporting 100-year-olds. The proportion of true responses would be predicted to be nearly zero.

Such calculations tell us that although Newman’s choices for *p* might look small in the abstract, they imply wildly implausibly high rates of misreporting at extreme ages. Copies of birth registration entries have been collected for all the Italians over the age of 110 in the data for Barbi and colleagues. To suggest, as Newman’s model does, that virtually every one of these individuals was born 10 years later than the date shown in his or her birth registration is not a credible suggestion.

A uniform civil registration system was established in Italy in the 1860s after Italian unification. It was well tested by the time the cohorts under study were born [3]. Birth certificates (“Atti di nascita”) were filled out by Civil Status Officers, well educated for their time, who entered births chronologically into bound volumes, a separate volume for each year in each municipality. For those over 110, the form of registration makes the kinds of clerical errors in years of birth assumed by Newman essentially impossible.

Newman goes on to vary the window of ages over which a log-linear function is fitted to hazards for the single cohort of Italian women born in 1904 from the Human Mortality Database. Hazard functions are well known to deviate from log-linear form beyond about the age of 80, so it hardly seems sensible to be calculating fits over ranges of ages where the model fails to fit.

No data set can be expected to be entirely free from error. All that Barbi and colleagues assert for their full data set is that “age misreporting is believed to be minimal in these data.” Occasionally, as in the anecdote that Newman supplies, a mistake will escape even a rigorous validation procedure. But the claims of Barbi and colleagues rest on nearly 4,000 carefully validated cases from an established registration system. A critique like Newman’s, which does not take specific features of these new data into account, can hardly carry force.
